# The effect of genetic bottlenecks and inbreeding on the incidence of two major autoimmune diseases in standard poodles, sebaceous adenitis and Addison’s disease

**DOI:** 10.1186/s40575-015-0026-5

**Published:** 2015-08-27

**Authors:** Niels C. Pedersen, Lynn Brucker, Natalie Green Tessier, Hongwei Liu, Maria Cecilia T. Penedo, Shayne Hughes, Anita Oberbauer, Ben Sacks

**Affiliations:** Center for Companion Animal Health, School of Veterinary Medicine, University of California, One Shields Avenue, 95616 Davis, CA USA; 1635 Grange Hall Road, 45432 Beavercreek, OH USA; 539 Ashland Ave, 14222 Buffalo, NY USA; Veterinary Genetics Laboratory, School of Veterinary Medicine, University of California, One Shields Avenue, 95616 Davis, CA USA; Department of Animal Science, College of Agricultural and Environmental Sciences, University of California, One Shields Avenue, 95616 Davis, CA USA

**Keywords:** Standard poodles, Genetic bottleneck, Inbreeding, Sebaceous adenitis, Addison’s disease

## Abstract

**Background:**

Sebaceous adenitis (SA) and Addison’s disease (AD) increased rapidly in incidence among Standard Poodles after the mid-twentieth century. Previous attempts to identify specific genetic causes using genome wide association studies and interrogation of the dog leukocyte antigen (DLA) region have been non-productive. However, such studies led us to hypothesize that positive selection for desired phenotypic traits that arose in the mid-twentieth century led to intense inbreeding and the inadvertent amplification of AD and SA associated traits.

**Results:**

This hypothesis was tested with genetic studies of 761 Standard, Miniature, and Miniature/Standard Poodle crosses from the USA, Canada and Europe, coupled with extensive pedigree analysis of thousands more dogs. Genome-wide diversity across the world-wide population was measured using a panel of 33 short tandem repeat (STR) loci. Allele frequency data were also used to determine the internal relatedness of individual dogs within the population as a whole. Assays based on linkage between STR genomic loci and DLA genes were used to identify class I and II haplotypes and disease associations. Genetic diversity statistics based on genomic STR markers indicated that Standard Poodles from North America and Europe were closely related and reasonably diverse across the breed. However, genetic diversity statistics, internal relatedness, principal coordinate analysis, and DLA haplotype frequencies showed a marked imbalance with 30 % of the diversity in 70 % of the dogs. Standard Poodles with SA and AD were strongly linked to this inbred population, with dogs suffering with SA being the most inbred. No single strong association was found between STR defined DLA class I or II haplotypes and SA or AD in the breed as a whole, although certain haplotypes present in a minority of the population appeared to confer moderate degrees of risk or protection against either or both diseases. Dogs possessing minor DLA class I haplotypes were half as likely to develop SA or AD as dogs with common haplotypes. Miniature/Standard Poodle crosses being used for outcrossing were more genetically diverse than Standard Poodles and genetically distinguishable across the genome and in the DLA class I and II region.

**Conclusions:**

Ancestral genetic polymorphisms responsible for SA and AD entered Standard Poodles through separate lineages, AD earlier and SA later, and were increasingly fixed by a period of close linebreeding that was related to popular bloodlines from the mid-twentieth century. This event has become known as the midcentury bottleneck or MCB. Sustained positive selection resulted in a marked imbalance in genetic diversity across the genome and in the DLA class I and II region. Both SA and AD were concentrated among the most inbred dogs, with genetic outliers being relatively disease free. No specific genetic markers other than those reflecting the degree of inbreeding were consistently associated with either disease. Standard Poodles as a whole remain genetically diverse, but steps should be taken to rebalance diversity using genetic outliers and if necessary, outcrosses to phenotypically similar but genetically distinct breeds.

## Lay summary

Standard Poodles suffer from a long list of autoimmune diseases including immune mediated hemolytic anemia, immune mediated thrombocytopenia, Evan’s syndrome, immune pancytopenia, chronic thyroiditis, temporal-mandibular myositis, and chronic active hepatitis. However, the two most vexing autoimmune disorders are sebaceous adenitis (SA) and Addison’s disease (AD). There has been a general belief that SA and AD entered the breed as a result of extensive inbreeding starting in the middle of the twentieth century that involved a small group of founders that produced show winning offspring. These offspring and their descendants were widely used by Standard Poodle breeders in North America and exported to the UK, Scandinavia, Australia Continental Europe. This artificial midcentury bottleneck (MCB) has created a severe imbalance and probable loss of genetic diversity. Using genetic tests based on 33 genome-wide and seven dog leukocyte antigen (DLA) class I and II short tandem repeat (STR) markers, we were able to study genetic diversity in poodles from the USA, Canada and Europe. Standard Poodles from all of these geographic regions were closely related, indicating a considerable ongoing transoceanic exchange of dogs. Although Standard Poodles still possess considerable total diversity, 70 % of this diversity resides in only 30 % of the population. This imbalance in diversity was both evident across the genome as well as in the DLA class I and II regions, the latter often associated with autoimmune disease. SA and AD entered the breed through different lines and at different times, but the traits underlying these diseases were more likely ancestral in many dog breeds and inadvertently concentrated as a result of the MCB. The DLA was not strongly implicated in either SA or AD, although several less common haplotypes conferred a moderate degree of risk or protection for one or the other disease, and dogs with minor DLA haplotypes were less prone to SA and AD than dogs with major haplotypes. It may be possible to re-distribute the genetic diversity in the breed with judicious mate selection based on genetic testing over a number of generations. Such an undertaking should reduce the incidence of deleterious simple recessive traits and complex genetic disorders such as autoimmune disease. The emphasis of mate selection should be on maximizing genetic differences by augmenting information gained from pedigrees with genetic tests that more accurately measure genetic diversity across the genome. This study also documented the current use of Miniature Poodles as outcrosses, which are genetically distinct and relatively free of SA and AD.

## Background

Canine autoimmune diseases occur in the same clinical forms as in humans and preferentially affect pure breeds [1]. The expression of autoimmune disease is influenced by genetic and non-genetic factors. Although measuring the influence of non-genetic factors is difficult, heritability studies for various autoimmune diseases in specific human populations, including dizygotic and monozygotic twins, range from 0.0008-1.0 with a median of 0.60 [2]. Limited studies in dogs also place the heritability of specific autoimmune disorders in the range of 0.50 to 0.60 [3–5]. Unlike numerous simple Mendelian traits that affect the health of dogs, a predisposition to autoimmune disease involves many genes and gene pathways [6–8].

The higher incidence of autoimmune disease in purebred compared to random-bred dogs mirrors what occurs in certain high risk human populations genetically restricted by race, ethnicity, and geography [9, 10]. Autoimmune disorders are also more likely to be reported in conformation than performance breeds and certain clinical forms occur across many breeds and others are more breed specific [3, 7, 11–15]. When studied, breeds of dogs that suffer the most from autoimmune disease also are often among the most inbred. Diminished genetic diversity has been associated with Pug dogs and necrotizing meningoencephalitis (NME), Italian Greyhounds and multiple autoimmune diseases [6], Nova Scotia Duck Tolling Retrievers with SLE-like disease [15], and Standard Poodles with sebaceous adenitis (SA) [7]. Inbreeding is an intrinsic problem of registered purebred dogs, because registry requirements preclude outcrossing. If diversity among the various founder populations is sufficient at the onset, it can be maintained indefinitely by judicious selection. However, genetic bottlenecks often complicate such efforts. Conformation, or show, breeding is particularly susceptible to artificial genetic bottlenecks compared to selection for performance traits [16].

The present study is concerned with the Standard Poodle, a breed that suffers from a number of autoimmune disorders, two of which, SA and Addison’s disease (AD) are particularly common. The breed has suffered a major artificial genetic bottleneck associated with show-winning bloodlines that rose to dominance in the 1950s. The history of this event was first described by Dr. John Armstrong in his description of the famous sire Sir Gay [17]. Although Sir Gay was not particularly noteworthy in the show ring, his claim to fame came from the breeding of his son Annsown Gay Knight of Arhill to Wycliffe Jacqueline of the Wycliffe Kennel. The mating of Annsown Gay Knight to Wycliffe Jacqueline produced dogs of show-winning form and their progeny were exported around the world and heavily used in close linebreeding to establish type. As a result, almost all subsequent show-winning Standard Poodle stud dogs have had Sir Gay in their pedigree. However, Wycliffe lines were only part of the genetic bottleneck and kennels such as Carillon, Lowmont, Puttencove, and Bel Tor also rose to prominence during this time, creating an even broader Midcentury bottleneck (MCB) [18]. The genetic contribution of these lines to contemporary Standard Poodles is referred to as % Wycliffe and % MCB. A third, and minor bottleneck, occurred at about this same time when Old English Apricot (OEA) poodles were used to improve the quality of dogs with the apricot coat color. The greatest % OEA is found in apricot and red Standard Poodles.

Even though multiple genetic associations on a number of chromosomes have been linked to autoimmune disease in humans, the strongest single genetic contributor of risk for autoimmune disease in people involves the human leukocyte antigen (HLA) complex and in particular certain class I and II polymorphisms [19, 20]. Similarly, most autoimmune diseases of dogs have demonstrated certain DLA class I [12] or II [6, 11, 14, 21, 22] genotypes or haplotypes that confer significantly increased risk. The strongest DLA class I risk (OR = 17) was demonstrated for pancreatic acinar atrophy in German Shepherd Dogs [12], while the strongest DLA class II associated risk (OR = 12.75) was shown for NME in Pug Dogs [23].

The hypotheses of this study is that inbreeding is an inherent risk factor for autoimmune disease in Standard Poodles and that significant risk associations will be identified in the regions on canine chromosome 12 that encode the DLA class I and II genes. In order to test these hypotheses, we elected to expand previous research that was done with Standard Poodles [7]. Therefore, the present study focused on the relationship between losses of genetic diversity, as determined by genome-wide and DLA class I and II-associated haplotype markers and pedigrees, with two common autoimmune conditions of the breed, SA and AD. Extensive and multi-generational pedigrees were also used to trace for common ancestors or lines that might be responsible for AD and SA.

## Results

### Assessment of genetic diversity based on 33 genomic STR markers

Allele frequencies for 33 genomic STR loci were determined for 761 dogs, including Standard Poodles from the USA, Canada and Europe, Miniature Poodles and Miniature/Standard Poodle crosses (Table 1). This data was then used to genetically assess the five populations (Table 2). Miniature and Miniature/Standard Poodle crosses were genetically more heterogeneous based on observed heterozygosity (Ho) than Standard Poodles, and expected heterozygosity (He) was somewhat lower indicating some substructure in the population due to non-random breeding. The value FIS, which is an inbreeding coefficient measuring the proportion of variance in the subpopulation contained in an individual, was slightly negative, indicating that Miniature Poodles and Miniature/Standard Poodle populations had individuals that were less related to each other than to dogs in the total population. However, this was a select population of genetically diverse Miniature Poodles that had been chosen for outcrossing and these values may not reflect all Miniature Poodles.

**Table 1:** Thirty three genomic STR loci, alleles and their overall frequencies in standard poodles from Europe (*n* = 57), USA (*n* = 478) and Canada (*n* = 138), and miniature poodles (*n* = 16) and Miniature/Standard Poodle crosses (*n* = 72). The *Canis familaris* autosome (CFA) location for each locus is provided

**INU055**	**AHT137**	**AHTH130**	**AHTh171-A**	**AHTh260**	**C22.279**	**FH2054**	**FH2848**	**INRA21**	**REN169D01**	**REN54P11**
**CFA10**	**CFA11**	**CFA36**	**CFA6**	**CFA16**	**CFA22**	**CFA12**	**CFA2**	**CFA21**	**CFA14**	**CFA18**
208(.001)	131(.238)	111(.018)	217(.008)	238(.571)	114(.001)	148(.011)	230(.003)	91(.373)	202(.008)	222(.003)
210(.176)	133(.008)	117(.002)	219(.362)	240(.026)	116(.085)	152(.028)	232(.012)	93(.002)	210(.001)^a^	226(.21)
212(.059)	135(.001)	119(.358)	221(.248)	242(.001)^a^	118(.438)	156(.556)	234(.013)	95(.425)	212(.06)	228(.205)
214(.342)	137(.172)	121(.14)	223(.008)	244(.04)	120(.006)	160(.014)	236(.034)	97(.039)	214(.004)	230(.003)
216(.378)	141(.349)	123(.11)	225(.177)	246(.22)	122(.001)	164(.004)	238(.113)	99(.05)	216(.394)	232(.353)
218(.028)	143(.017)	127(.117)	227(.009)	248(.071)	124(.355)	168(.298)	240(.759)	101(.089)	218(.305)	234(.212)
220(.013)	145(.038)	129(.213)	229(.028)	250(.021)	126(.033)	172(.079)	242(.065)	103(.008)	220(.001)	236(.006)
222(.002)^a^	147(.067)	131(.02)	231(.003)	252(.039)	128(.025)	176(.009)	244(.001)	105(.013)	222(.032)	238(.007)
	149(.008)	133(.013)	233(.003)	254(.004)	130(.056)	180(.001)		109(.001)	224(.18)	242(.001)
	151(.102)	135(.009)^a^	235(.135)	256(.006)					226(.016)	
			237(.021)							
**AHTk211**	**INU005**	**INU030**	**REN169O18**	**LEI004**	**REN105L03**	**REN162C04**	**AHTk253**	**FH2001**	**REN247M23**	**REN64E19**
**CFA26**	**CFA33**	**CFA12**	**CFA29**	**CFA37**	**CFA11**	**CFA2**	**CFA23**	**CFA23**	**CFA15**	**CFA34**
87(.16)	110(.022)	144(.316)	156(.001)	85(.707)	227(.014)	200(.021)	280(.016)	124(.007)	266(.041)	139(.001)
89(.042)	124(.526)	146(.168)	160(.026)	95(.094)	231(.24)	202(.173)	284(.157)	132(.443)	268(.481)	143(.01)
91(.66)	126(.431)	148(.064)	162(.594)	107(.17)	233(.175)	204(.057)	286(.085)	136(.043)	270(.265)	145(.432)
93(.002)^a^	128(.007)	150(.085)	164(.303)	109(.029)	235(.003)	206(.569)	288(.399)	140(.019)	272(.209)	147(.27)
95(.136)	130(.009)	152(.365)	166(.01)		237(.014)	208(.082)	290(.225)	144(.273)	278(.004)	149(.032)
	138(.005)	156(.002)	168(.034)		239(.012)	210(.063)	292(.119)	148(.207)		153(.244)
			170(.026)		241(.542)	212(.035)		152(.008)		155(.01)
			172(.006)		243(.001)					
**AHT121**	**VGL0760**	**VGL0910**	**VGL1063**	**VGL1165**	**VGL1828**	**VGL2009**	**VGL2409**	**VGL2918**	**VGL3008**	**VGL3235**
**CFA13**	**CFA7**	**CFA9**	**CFA10**	**CFA11**	**CFA18**	**CFA20**	**CFA24**	**CFA29**	**CFA30**	**CFA32**
92(.019)	12(.324)	12(.001)	8(.007)	14(.001)^a^	15(.001)	10(.002)	13(.028)	12(.001)	12(.001)	12(.15)
94(.028)	13(.001)	13(.046)	9(.004)^a^	15(.002)	16(.061)	11(.058)	14(.254)	13(.099)	13(.01)	13(.05)
96(.011)	14(.013)	14(.004)	10(.001)	16(.049)	17(.022)	12(.03)	15(.235)	14(.202)	14(.038)	14(.14)
98(.341)	15(.018)	15(.006)	11(.003)	17(.001)	18(.093)	13(.245)	16(.127)	15(.17)	15(.22)	15(.026)
100(.092)	19(.088)	15.1(.004)	12(.019)	18(.016)	19(.478)	14(.236)	17(.181)	16(.038)	16(.03)	16(.336)
102(.004)	19.2(.132)	16.1(.003)	13(.181)	19(.008)^a^	20(.269)	15(.042)	18(.13)	16.3(.001)	17(.562)	17(.222)
104(.161)	20(.022)	17.1(.128)	14(.13)	20(.001)	21(.047)	9(.388)	19(.043)	17(.004)	18(.021)	18(.074)
106(.076)	20.2(.18)	18.1(.289)	15(.051)	21(.078)	22(.008)		20(.001)^a^	17.3(.043)	18.2(.001)	
108(.203)	21.2(.05)	19(.001)	16(.116)	22(.002)				18.3(.02)	19(.1)	
110(.044)	22.2(.013)	19.1(.147)	17(.029)	23(.003)				19.3(.11)	20(.015)	
112(.019)	23.2(.086)	20.1(.026)	18(.047)	24(.021)				20.3(.102)	21(.003)	
114(.001)	24.2(.061)	21.1(.278)	19(.359)	25(.096)				21.3(.154)		
	25.2(.01)	22(.003)	20(.024)	26(.469)				22.3(.051)		
	26.2(.001)^a^	22.1(.019)	21(.017)	27(.128)				23.3(.004)		
		23(.038)	22(.004)	28(.117)						
		23.1(.006)	23(.006)	29(.004)						
		24(.001)	24(.001)	30(.003)^a^						
				31(.001)^a^						
				32(.001)^a^						
				34(.001)^a^						

^a^indicate alleles found only in Miniature Poodles

**Table 2:** Genetic assessment of standard poodles from Europe, USA and Canada

			# Alleles/Locus				
	# Dogs		Total	Effective	Ho	He	FIS
Europe SP	57	Mean	6.727	3.443	0.662	0.673	0.016
		SE	0.332	0.209	0.021	0.020	0.015
USA SP	478	Mean	8.061	3.384	0.641	0.668	0.041
		SE	0.403	0.201	0.021	0.021	0.006
Canada SP	138	Mean	7.152	3.360	0.648	0.665	0.024
		SE	0.359	0.217	0.021	0.020	0.011
Total SP	673	Mean	8.333	3.413	0.644	0.671	0.04
MiniP	16	Mean	6.485	4.069	0.744	0.733	−0.021
		SE	0.357	0.227	0.021	0.013	0.028
MiniP/SP	72	Mean	7.030	3.726	0.746	0.706	−0.054

Miniature Poodles (*n* = 16) used for outcrossing and Miniature/Standard Poodle crosses (*n* = 72) were also included. In addition to the total and effective number of alleles at each locus, genetic assessment included observed homozygosity (Ho), expected heterozygosity (He) and FIS as a measure of outbreeding (negative value) or inbreeding (positive value) within the population

The three Standard Poodle populations were remarkably similar in terms of Ho. However, He was higher than Ho for Standard Poodles from all three geographic locations, although somewhat greater for Standard Poodles from the USA than from Canada and Europe. The resulting positive values for FIS in Standard Poodle indicated that population substructure involving more inbred dogs existed in all three groups. The mean number of alleles per locus in dogs from different regions differed, but this appeared to be a result of sample size, because the number of effective alleles was similar among dogs from the three geographic areas (Table 2).

### Internal relatedness among standard poodles

**Fig. 1:**
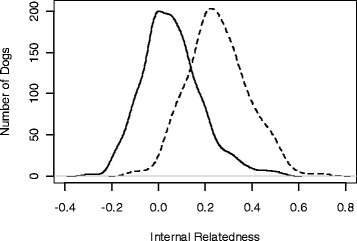
Distribution of IR estimates in 664 Standard Poodles based on intra-breed diversity (solid line), compared with IR adjusted for diversity lost during breed evolution (dashed line). The loss of diversity was determined by comparing allele frequencies at the same loci between Standard Poodles and randomly breeding village dogs from the Middle East, SE Asia, and islands and nations of the Pacific [16]

Internal relatedness (IR) based on 33 genomic STRs was calculated for 664 of the 673 Standard Poodles in the study (Fig. 1). The average IR value for the population was around 0.0 and only a small proportion had IR values approaching 0.25, which would be equivalent to offspring of full-sibling parents. Individual IR values were then adjusted for diversity that was likely to have been lost during the evolution of the breed using allele and allele frequency data of 533 randomly mating indigenous village dogs from Lebanon, Iran, Taiwan, Thailand, Philippines, Brunei, Cook Islands and Bali [16]. This population contains much of the collective genetic diversity of modern breeds [16, 42]. Adjusted IR values were shifted to the right with the average adjusted IR closer to 0.25 (Fig. 1). Therefore, average Standard Poodles in this study were interrelated to the same level as offspring of full sibling village dog parents.

### Internal relatedness among standard poodles with SA or AD

**Fig. 2:**
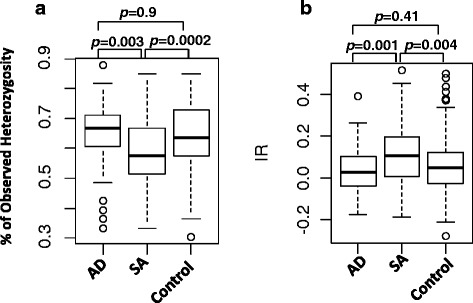
Comparison of % Observed Heterozygosity (**a**) and IR (**b**) based on 33 STRs among Addison (*n* = 74), SA (*n* = 61) and Control (*n* = 314) Standard Poodles from USA. Data were analyzed with one-way ANOVA

In order to study the effect of inbreeding on the incidence of SA and AD, % observed heterozygosity and IR values based on 33 STRs of healthy Standard Poodles (*n* = 314) and Standard Poodles suffering from AD (*n* = 74) or SA (*n* = 61) were compared (Fig. 2). The three groups of dogs were only from the USA, as disease records for Poodles from Europe and Canada were incomplete. The two different indices of heterozygosity yielded somewhat different results. Based on average Ho, there appeared to be some degree of differentiation between healthy, SA and AD populations (Fig. 2a). Healthy control dogs and dogs with AD appeared to be genetically indistinguishable, whereas dogs with SA had significantly lower average heterozygosity than either of the other populations. In order to see how these population differences might relate to inbreeding, IR values were compared between the three populations (Fig. 2b). No difference in the degree of inbreeding was observed between dogs with AD and healthy control dogs. However, dogs with SA were significantly more inbred than healthy dogs or dogs with AD.

### Principal coordinate analysis (PCoA)

**Fig. 3:**
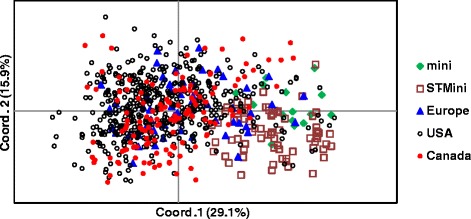
PCoA plot based on 33 genomic STRS of 761 poodles. The poodles were from various regions of the world and included mainly purebred Standard Poodles, with a smaller number of Miniature Poodles (*n* = 16) and Standard Poodle/Miniature Poodle crosses (*n* = 72). The bulk of purebred Standard Poodles from Europe, Canada and the USA were clustered together with some genetic outliers from all regions. Miniature Poodles were clearly distinct from the general population of Standard Poodles. Standard Poodles that had been outcrossed in the past to Miniature Poodles formed an outlier population

A majority of Standard Poodles were clustered around and to the left of the coordinate 1 and 2 axis of a PCoA plot, while most genetic outliers were positioned mostly to the right of the main population along with the Miniature Poodles and the Miniature/Standard Poodle crosses (Fig. 3). There was very little genetic differentiation between Standard Poodles from the USA, Canada and Europe.

**Fig. 4:**
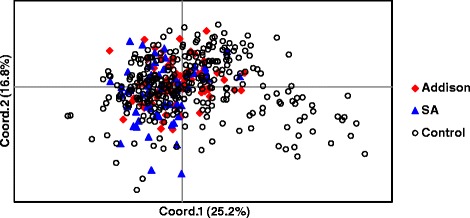
PCoA plot of Standard Poodles showing the genetic relationships between healthy dogs (*n* = 314) and dogs suffering from either SA (*n* = 61) or AD (*n* = 74). Diseased case and healthy control dogs were only from the USA

The distribution of Standard Poodles with SA and AD, and healthy controls was also examined by PCoA (Fig. 4). There were insufficient confirmed case and controls from the European population to be analyzed. American Standard Poodles suffering from AD and SA were indistinguishable from the majority of the control population, which tended to form a tight cluster just to the left of the coordinate 1 and 2 axis. This cluster coincided with the main body of Standard Poodles shown in Fig. 3. Outliers tended to be free of both SA and AD.

### DLA class I and II haplotypes as determined by linked STR loci

Forty-three STR-associated DLA class I haplotypes were identified among Standard, Miniature and Miniature/Standard Poodle crosses (Table 3). Eight of these haplotypes were found only in Miniature Poodles and crosses. Four haplotypes, 1001–1004, comprised 1073/1522 (70.5 %) of the total haplotypes that were identified (Table 3). Twenty-eight STR-associated DLA class II haplotypes were identified and five of these were found only in Miniature Poodles and crosses (Table 3). Two of these haplotypes, 2001 and 2002, made up over two thirds of the total haplotypes in the population. STR-associated DLA class II haplotypes, when tested, corresponded to known DLA-DRB1/DQA1/DQB1 haplotypes determined by exon 2 sequencing (Table 3).

**Table 3:** DLA STR-based class I and II haplotypes and their frequencies in standard poodles (*n* = 673), Miniature Poodles (*n* = 16), and miniature/standard poodle crosses (72)

Class I			Class II			
ID	Haplotype	Frequency	ID	Haplotype	Frequency	DLA-DRB1/DQA1/DQB1
1001	380/373/281/182	0.259	2001	343/324/284	0.605	01501/00601/02301
1002	380/365/281/181	0.200	2002	343/327/280	0.091	01501/00901/00101
1003	387/375/277/186	0.157	2003	343/324/282	0.092	01503/00601/02301
1004	393/379/277/183	0.090	2004	351/327/268	0.027	02001/00401/01303
1005	389/371/277/181	0.056	2005^b^	339/322/280	0.024	00101/00101/00201
						00901/00101/008011
1006	387/375/293/180	0.043	2006	339/325/280	0.032	01502/00601/02301
1007	380/372/281/182	0.031	2007	351/327/280	0.016	02001/00401/01303
1008	386/373/289/182	0.018	2008	339/327/276	0.011	00101/00101/03601
1009	382/377/277/184	0.011	2009	351/324/280	0.016	
1010	384/371/277/186	0.016	2010	345/329/280	0.016	01201/00401/013017
1011	376/365/281/180	0.020	2011^c^	345/322/284	0.020	00901/00101/008011
1012	388/369/289/188	0.011	2012^c^	345/322/280	0.008	00901/00101/008011
1013	392/373/289/186	0.010	2013	345/327/284	0.008	00201/00901/00101
1014	375/373/287/178	0.008	2014	339/322/284	0.005	
1015	380/373/291/186	0.003	2015^a^	339/327/280	0.005	
1016	382/371/277/178	0.005	2016^c^	339/323/284	0.003	00601/05011/00701
1017	386/373/289/178	0.005	2017	343/322/280	0.004	
1018^a^	375/373/287/186	0.013	2018^c^	339/324/284	0.001	00601/05011/00701
1019	380/373/287/185	0.003	2019	345/324/284	0.001	
1020^a^	388/369/289/184	0.004	2020	349/324/284	0.001	
1021	380/373/289/186	0.003	2021^a^	339/324/268	0.001	
1022	380/375/281/181	0.001	2022	339/327/282	0.001	
1023	380/379/281/181	0.001	2023	341/323/282	0.006	
1024	387/373/281/182	0.001	2024^a^	343/323/280	0.001	
1026	390/369/289/186	0.001	2025^a^	351/321/280	0.002	
1027	391/371/277/181	0.001	2026	351/324/284	0.001	
1028^a^	376/369/291/186	0.001	2027	343/325/284	0.001	
1029	380/365/281/182	0.003	2028^a^	345/327/288	0.001	
1030	380/373/293/178	0.006				
1031^a^	382/371/277/186	0.003				
1032^a^	382/377/277/178	0.001				
1033^a^	382/379/277/181	0.001				
1034^a^	382/379/277/182	0.001				
1035	386/373/277/184	0.001				
1036^a^	389/365/289/180	0.002				
1039	379/364/281/181	0.001				
1040^a^	380/371/277/186	0.001				
1041	386/374/277/186	0.001				
1042	389/375/293/186	0.001				
1043	393/381/277/183	0.001				
1045	376/371/277/186	0.001				
1046	376/379/291/180	0.001				
1057	387/376/277/186	0.001				

^a^Associated with Miniature Poodles

^b^Single STR haplotype associated with two known exon 2 sequence based haplotypes

^c^Two different STR haplotypes associated with the same exon 2 sequence based haplotype

Specific class I and II haplotypes tended to form extended haplotypes over a region of approximately 2 Mb. Recombination was reflected by new linkages between class I and II haplotypes, forming 49 extended haplotypes, the five most common being 1001/2001, 1002/2001, 1003/2001, 1004/2002, 1005/2003 (data not shown). Homozygosity of STR-associated DLA class I haplotypes was always associated with homozygosity in linked class II haplotypes, reflecting the strong linkage disequilibrium in the DLA. Simple mutations in a single STR allele also led to uncommon situations where the same class II STR-associated haplotypes corresponded to two published exon 2 sequence based haplotypes (Table 3). Conversely, two STR-associated haplotype was also found to be in linkage with the same sequence based haplotypes (Table 3). These situations can be explained by the much higher mutation rate of STRs compared to SNPs [24].

### DLA class I/II haplotype associations with SA and AD

The frequency of DLA class I based on STRs in healthy, SA and AD affected, subpopulations of Standard Poodles from the USA was compared (Table 4). There were insufficient data on the health status of dogs from outside the USA to include them in the comparison. Among all of the STR associated DLA class I haplotypes, only 1003 was significantly associated with disease. The relative risk (RR) for SA with dogs having the 1003 haplotype was 1.63 (95 % CI 1.13-2.33, z statistic 2.63, *p* = 0.0085). The RR for AD was 1.43 (95 % CI 1.0-2.05, z statistic 1.954, *p* = 0.0507). There was an impression that dogs with less common class I haplotypes were at a significantly reduced risk of developing SA or AD. To test this possibility, class I haplotypes were divided into two groups, 1001–1007 and 1008–1057. The RR for SA in dogs with minor haplotypes was 0.428 (95 % CI 0.214-0.859, z statistic 2.388, *p* = 0.0169). The RR for AD among dogs possessing the minor class I haplotypes was 0.53 (95 % CI 0.298-0.84, z statistic 2.17, *p* = 0.03). Therefore, dogs with minor class I haplotypes were 2.34 times less likely to develop SA and 1.89 times less likely to develop AD than the control population.

**Table 4:** Number and frequency of DLA class I haplotypes in 314 healthy control standard poodles from the USA and cohorts suffering from AD (*n* = 74) or SA (*n* = 61). Number of haplotypes = 2x number of dogs

Haplotype	AD	SA	Control
1001	48(0.32)	30(0.25)	164(0.26)
1002	28(0.19)	27(0.22)	131(0.21)
1003	32(0.22)	30(0.25)	95(0.15)
1004	70.05)	8(0.07)	41(0.07)
1005	6(0.04)	3(0.03)	47(0.08)
1006	4(0.03)	11(0.09)	33(0.05)
1007	110.07)	5(0.04)	21(0.03)
1008	0	0	16(0.03)
1009	0	0	15(0.02)
1010	0	0	10(0.02)
1011	3(0.02)	2(0.02)	15(0.02)
1012	3(.02)	2(.02)	1(.002)
1013	0	0	6(0.01)
1014	0	0	6(0.01)
1015	0	0	2(0.003)
1016	0	0	7(0.01)
1017	1(0.007)	0	4(0.006)
1019	0	0	5(0.008)
1021	0	0	1(0.002)
1023	0	0	1(0.002)
1024	0	1(0.008)	0
1026	0	1(0.008)	0
1029	1(0.007)	0	2(0.003)
1030	1(0.007)	0	3(0.005)
1039	0	0	1(0.002)
1041	0	0	1(.002)
1042	0	2(0.02)	0
1045	2(0.01)	0	0
1057	1(0.007)	0	0
Total	148	122	628

The DLA class II haplotypes were also tested for disease associations (Table 5). Dogs with haplotype 2004 were 2.8 times more likely to develop SA (*p* = 0.009) and dogs with haplotype 2006 were 2.4 times more likely to develop AD (*p* = 0.01). However, dogs possessing haplotypes 2004 and 2006 made up only 6 % of the total population (Table 3).

**Table 5:** Number and frequency of DLA class II haplotypes in 314 healthy control standard poodles from the USA and cohorts suffering from AD (*n* = 74) or SA (*n* = 61). Number of haplotypes = 2x number of dogs

Haplotype	AD	SA	Control
2001	109(0.74)	87(0.71)	384(0.61)
2002	7(0.05)	8(0.07)	41(0.07)
2003	9(0.06)	5(0.04)	54(0.09)
2004	3(0.02)	9(0.07)	16(0.03)
2005	1(.007)	0	20(.03)
2006	12(0.08)	5(0.04)	21(0.03)
2007	1(0.007)	2(0.016)	17(0.03)
2008	0	0	15(.024)
2009	0	1(0.008)	8(0.013)
2010	0	0	10(0.016)
2011	3(0.02)	2(0.016)	15(0.024)
2012	2(0.014)	1(0.008)	2(0.003)
2013	0	0	6(0.01)
2014	0	0	7(0.01)
2016	0	0	4(0.006)
2017	0	0	1(0.002)
2018	0	0	1(0.002)
2019	0	0	1(0.002)
2020	0	2(0.016)	0
2023	1(0.007)	0	3(0.005)
2026	0	0	0
2027	0	0	2(0.003)
Total	148	122	628

The frequency of homozygous DLA class I and II haplotypes in control dogs was compared to that of American Standard Poodles suffering from either SA or AD (Table 6). Homozygosity in either DLA class I or II regions was not a significant risk factor for either SA or AD.

**Table 6:** The frequency of homozygous STR-associated DLA class I and II haplotypes in healthy (control) dogs compared to dogs suffering from SA or AD (cases)

	AD		SA	
	Class I	Class II	Class I	Class II
Case	0.24	0.53	0.12	0.51
Control	0.17	0.44	0.17	0.44
RR	1.4	1.2	0.67	1.16
*p-value*	0.15	0.15	0.28	0.3

### Midcentury bottleneck (MCB), Wycliffe and Old English apricot (OEA)

Standard Poodles from the 1950s onward can be ranked for the genetic influence of MCB founders, the Wycliffe line, and Old English Apricots using a database that presently contains pedigrees for 235,351 dogs (Table 7). The average % MCB for 203,397 of these dogs with complete pedigrees back to the MCB was 4.6 in the decade of the 1950s, but then rose rapidly to a peak of 55.5 % in the 1990s, followed by a plateau through the 2000s and a slight decrease to 51.7 % in the 2010s. This was paralleled by the percentage contributions of the Wycliffe line, although this was consistently 5-9 % less than MCB. The average %OEA was 0.46 in the 1950s and then rose rapidly to 5.0 % in the 1960s, and then plateaued at 5.1–8.3 % through the 2010s.

**Table 7:** The average contribution of MCB, Wycliffe, and OEA ancestry to standard poodles during the decades of the 1950s and into the 2010s

Decade	Disease	# Dogs	% MCB	% Wycliffe	% OEA	AvgCOI-10	AvgCOI-15
1950	AD	0	0.0	0.0	0.0	0.0	0.0
1950	SA	0	0.0	0.0	0.0	0.0	0.0
1950	Total population	7,371	4.6	2.0	0.5	9.2	9.6
1960	AD	0	0.0	0.0	0.0	0.0	0.0
1960	SA	12	69.1	61.6	0.0	16.3	17.7
1960	Total population	9,541	18.5	13.0	5.1	11.6	12.7
1970	AD	4	84.8	79.6	0.0	24.5	27.4
1970	SA	17	76.3	70.3	0.0	23.2	26.1
1970	Total population	16,541	37.7	30.8	6.9	12.9	15.3
1980	AD	34	69.8	59.2	2.7	20.6	24.9
1980	SA	142	70.8	62.6	0.8	22.1	26.2
1980	Total population	25,549	52.7	44.5	6.0	15.1	19.0
1990	AD	209	57.7	49.2	7.0	14.2	19.4
1990	SA	204	61.4	52.8	4.3	15.7	20.9
1990	Total population	48,080	55.5	46.8	6.2	13.5	18.6
2000	AD	316	55.6	47.3	7.4	10.8	17.0
2000	SA	149	60.1	50.3	3.6	11.2	17.9
2000	Total population	71937	53.9	45.1	7.0	9.4	15.6
2010	AD	23	53.0	44.7	8.5	6.8	13.7
2010	SA	11	58.3	49.2	5.4	11.6	19.0
2010	Total population	24378	51.7	43.4	8.3	6.6	13.4

The % MCB, % Wycliffe and % OEA were calculated from the Standard Poodle Database during each decade as well as for dogs known to have had SA and AD. The average COI over 10 and 15 generations were also determined for dogs born in each decade. Data on SA and AD represent cases voluntarily reported to the Standard Poodle health registry database [25] and not actual incidence. Data for the 2010s were only through 2014

The average coefficients of inbreeding (COI) were also calculated over 10 and 15 generations for dogs registered during the decades between the 1950s and 2010s (Table 7). The average COI for 10 generation pedigrees rose from 9.2 in the 1950s to 15 % in the 1980s in parallel with a dramatic rise in the % MCB and % Wycliffe, followed by a decline to 6.6 % in the first half of the 2010s. Analysis of 15 rather than 10 generation pedigrees demonstrated parallel but higher average coefficients of inbreeding, rising from 9.6 % in the 1950s to 19 % in the 1980s and 1990s, followed by a decline to 13.4 % among dogs tested so far in the 2010s. The more than two times higher COIs calculated from the 15 compared to 10 generation pedigrees more accurately reflected the greater number of common ancestors present in earlier generations and the influence of the MCB on subsequent breed diversity.

### Relationship of MCB, Wycliffe and OEA ancestry to SA and AD

Reports of SA first appeared in the Standard Poodle database in the 1960s and rose progressively through the 1980s, plateaued through the 2000s, and were possibly decreasing in the 2010s (Table 7). AD was not reported in the registries until the 1970s and reached a peak during the 2000s (Table 7). Both diseases rose in incidence with the % Wycliffe and % MCB in the population. Percent OEA was not related to either SA or AD. The increased contribution of Wycliffe and MCB correlated with a rise in breed-wide COI (Table 7). The average COI for dogs with SA in the 1960s, when it was first recognized, was 17.7 %, compared to 12.7 % for the total population. The COIs for dogs with SA rose to around 26 % in the 1970s and 1980s, followed by a slow decline to 19 % in the 2010s. The COIs for the total population also rose progressively during this same period, paralleling the SA population but from 1–11 % lower. The average COI for dogs with AD was 27.4 % in the 1970s, when first being reported, compared to 15.3 % for the total population. It then declined for dogs with AD to 17 % in the 2000s and 13.7 % in the 2010s, not different from the total population. The average COIs for dogs with SA and AD were not appreciably different from each other during the 1970s to the present. The equilibration of COIs between dogs with SA and AD and the population as a whole reflected a return to more random breeding practices following the extremes of the MCB .

### Influential ancestors of contemporary standard poodles

**Fig. 5:**
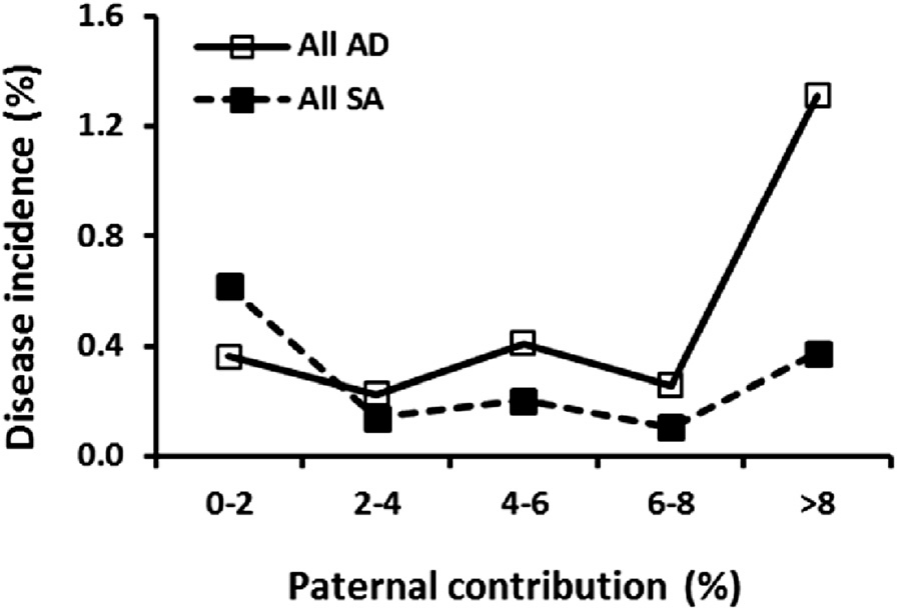
A comparison of the relationship coefficient ( % ancestry) of Berkham Hansel of Rettats in an individual pedigree and the ratio of AD (*n* = 512) to healthy Standard Poodles (*n* = 1643). Berkham Hansel was born in 1937 and was the ninth most influential ancestor for dogs suffering from AD

**Fig. 6:**
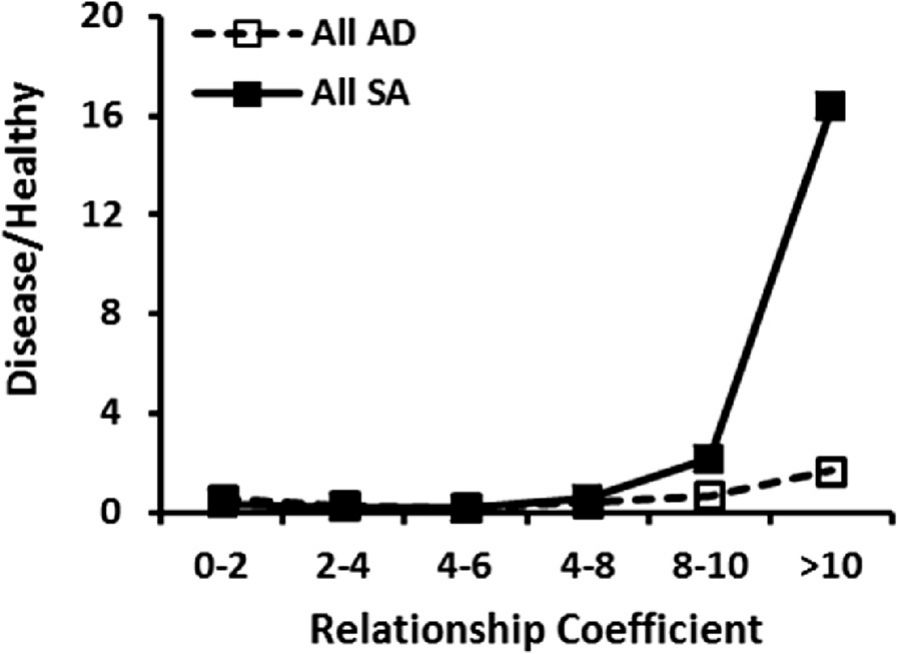
A comparison of the relationship coefficient ( % ancestry) of Mogene’s Beauzeaux in an individual pedigree and the ratio of SA (*n* = 465) to healthy Standard Poodles (*n* = 1643). Mogene’s Beauzeaux was born in 1962 and is the sixth most influential ancestor for dogs suffering from SA

**Fig. 7:**
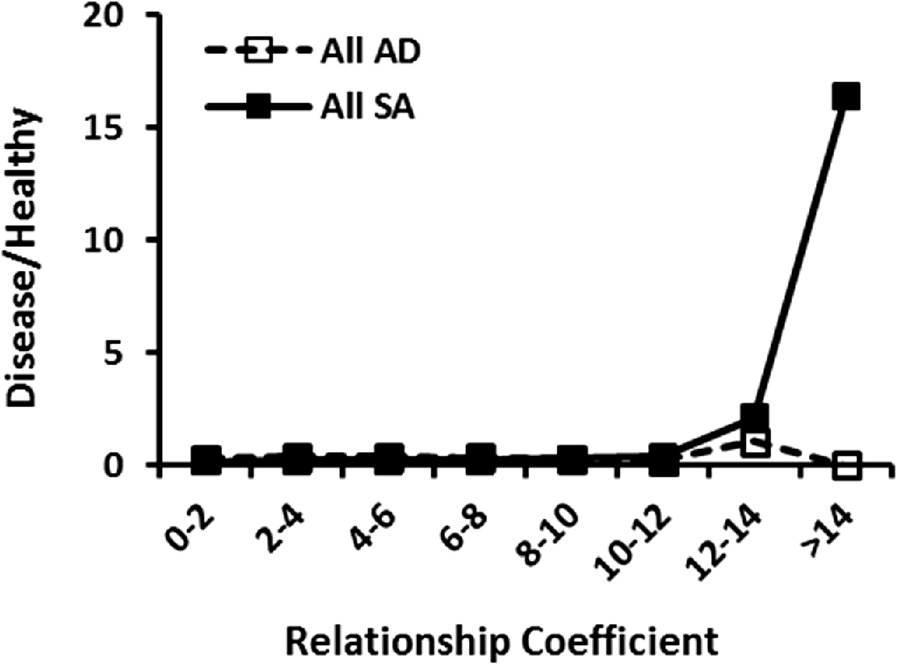
A comparison of the relationship coefficient ( % ancestry) of Haus Sachse’s Rebecca in an individual pedigree and the ratio of SA (*n* = 465) to healthy Standard Poodles (*n* = 1643). Haus Sachse’s Rebecca was born in 1964 in a bloodline separate from Mogene’s Beauzeaux and is the tenth most influential ancestor for dogs suffering from SA

The Poodle Health Registry data base [25], which contains voluntary information, was coupled with private and solicited communications to identify three different populations from among 235,351 pedigrees: 1) healthy dogs with no autoimmune disease in at least three generations, *n* = 1,643; 2) dogs with AD, *n* = 512; and 3) dogs with SA, *n* = 465. The top 26 most influential ancestors were calculated for each population (Table 8). Twenty-three most influential ancestors of healthy dogs appeared on the most influential ancestor list for dogs with AD, with two dogs, Berkham Hansel of Rettats and Whippendell Lolita, absent from the ancestors of healthy and SA dogs. Twenty one ancestors of healthy dogs were also present on the list of most influential ancestors of dogs with SA, and three others, Mogene’s Beauzeaux, Haus Sachse’s Rebecca and Aliyah Desperado, were not found among the ancestors of healthy and AD dogs. The contribution of the five unique ancestors to either SA or AD was then determined by comparing the ratio of diseased to healthy dogs to the contribution of each unique ancestor to their pedigrees (Table 8, Figs. 5, 6 and 7). When the percentage contribution of Berkham Hansel of Rettats to a pedigree rose to a certain threshold level, the incidence of AD rapidly increased (Fig. 5). The same was found true for Mogene’s Beauzeaux and Haus Sachse’s Rebecca for SA (Figs. 6, 7).

**Table 8:** Ranking of most influential ancestors of healthy dogs, dogs with SA and dogs with AD

Registered name	Healthy	AD	SA	Sex	DOB
Wycliffe Jacqueline	1	1	1	F	04/03/1954
Annsown Gay Knight of Arhill	2	2	2	M	10/12/1955
Annsown Sir Gay	3	3	3	M	14/03/1949
Prinz Alexander von Rodelheim	4	5	4	M	30/06/1925
Whippendell Carillon	5	4	5	M	15/05/1923
Whippendell Gaspard	6	6	8	M	10/09/1916
Anderl vom Hugelberg	7	7	7	M	22/07/1923
Blakeen Cyrano	8	8	9	M	28/07/1934
Nymphaea Swift	9	12	12	M	22/06/1926
Leonore von der Seestadt	10	10	11	F	04/04/1925
Meta	11	13	15	F	01/07/1921
Lidia vom Feuerbachtal	12	11	13	F	24/05/1927
Nelly von der Schneeflocke of Blakeen	13	14	14	F	29/01/1929
Derian Diana	14	18	18	F	06/02/1926
Chloe (#2)	15	17	19	F	
Whippendell Mascotterina	16	15	20	F	21/04/1914
Mira Labory	17	19	21	F	21/04/1926
Harpendale Lady Teazle	18			F	19/02/1920
Santo-Labory of Carillon	19	16	17	M	14/06/1948
Quality of Piperscroft	20	21		F	28/07/1933
Jose of Kyles	21			F	01/01/1921
Scarletts Gillian	22	22		F	13/07/1928
Whippendell Drapeau	23	23		M	08/02/1920
Vulcan Champagne Wopper	24	20		M	19/06/1950
Whippendell Cordon Bleu	25			M	24/07/1909
Carina von Sadowa of Blakeen	26	25		F	16/07/1934
Berkham Hansel of Rettats		9		M	20/12/1937
Whippendell Lolita		24		F	30/03/1926
Mogene’s Beauzeaux			6	F	20/10/1962
Haus Sachse’s Rebecca			10	F	06/05/1964
Aliyah Desperado			16	M	23/09/1977

## Discussion

The objective of this study was to determine the influence of inbreeding and specific STR-associated DLA class I and II haplotypes on the incidence of two common autoimmune diseases in Standard Poodles, SA and AD. As such, it is essentially the story of the MCB and how events in both North America and Europe during and after WWII have influenced genetic diversity and certain health problems in Standard Poodles around the world. The study used both pedigrees and DNA-based genetic markers across the genome and in the DLA class I and II regions to investigate the effects of the MCB on genetic diversity and on health. Classical genetic measurements such as Ho and He for Standard Poodles were comparable to those of many other pure breeds [24], but lower than outbred Landrace-type dogs such as Jack Russell Terriers [24] and indigenous village dogs from the Middle East, SE Asia and Pacific regions [16, 23, 26]. Standard Poodles from the USA, Canada and Europe showed minor genetic differences, supporting a considerable transoceanic exchange of dogs. These findings confirmed observations reported in an earlier study of Standard Poodles from these regions [7]. In order to better define genetic diversity, allele numbers and frequency data was used to compute IR values and construct PCoA plots. DLA class I and II haplotype frequencies were used to interrogate diversity in the DLA and risk associations with SA and AD, while pedigrees were used to better define the evolution of the MCB and its effect on genetic diversity and disease.

IR has been widely used in studies that have investigated the relationship between inbreeding and fitness. For instance, studies have shown fitness (reproduction, survival) to be inversely correlated to IR in long-finned pilot whales, grey seals and wandering albatross [27]. Sea lions with high IR values were more likely to suffer from a variety of infections than healthy animals that had suffered a sudden traumatic death [28]. IR was also linked to greater reproductive success in male Antarctic fur seals [29] and improved pre-weaning pup survival in grey seals [30]. Banks and colleagues [31] used IR values based on nine genomic STR markers, as well as a STR loci linked to *DAB1* of the major histocompatibility complex, to study the role of heterozygosity on survival and endoparasitism of mountain brushtail possums in Australia. Forstmeier and colleagues [32] demonstrated a positive correlation between lack of heterozygosity as measured by IR and fitness related traits in zebra finches. Remarkably, a small panel of 11 STRs in the latter study produced equally strong results as the large panel of 1359 SNP markers, supporting the continuing value of STRs in population genetics in an era dominated by SNPs.

A genetic profile of Standard Poodles from across the world was created using IR as a measure of heterozygosity and inbreeding. The average Standard Poodles appeared to be reasonably unrelated based on IR values from across the breed. However, when the IR curve was adjusted for diversity lost during breed evolution using village dog allele frequencies as a gold standard [16], average contemporary Standard Poodles were more akin to offspring of village dog parents that were full siblings. This loss of breed diversity has probably occurred in both slow and sudden steps. A “natural” loss of diversity undoubtedly started even earlier when the forefathers of the breed began to deviate from the native (landrace-type) dogs of their regions in Europe as they took on more human selected form and function. The next artificial genetic bottleneck occurred in 1874 in England when the breed registry was established. However, the founding population was apparently quite large and involved dogs with a wide range of form and function and from several geographic regions. It can be surmised that the original registered poodles possessed considerable genetic diversity and that the greatest loss of diversity occurred over the following century. WWII caused many European bloodlines to disappear, especially in countries such as England and Germany where Standard Poodles were prominent. Size limits were also subsequently applied by some registries in Europe, eliminating bloodlines with dogs exceeding the size standard. The loss of breeding lines and ease of international travel also encouraged an influx of Standard Poodles from North America, which were products of the MCB and a better fit to the desired breed type of the time. The fall of the Iron Curtin had a similar effect, but rather than providing a rich source of diversity that had been long insulated from the rest of Europe and North America, breeders from the Iron Curtin countries rushed to replace their indigenous bloodlines with what they perceived as more elegant Western dogs.

Although pedigrees of sufficient depth are helpful in documenting the various artificial genetic bottlenecks of the last 150 years, they do not provide an accurate picture of how these events have affected genetic diversity. Therefore, the purpose of this study was to combine pedigrees and modern DNA-based tests to better define how much the breed has been genetically affected by past events, and in particular the MCB. The effective number of alleles per genomic locus for the breed was low (3.4 alleles/locus) and, on average, 70 % of Standard Poodles tended to share the same two alleles at each locus. This was a portion of what was found in many indigenous village dog populations [16]. This loss of diversity is also reflected by numbers of published maternal and paternal lineages. Standard Poodles descend from one major (82 % of individuals) and 4 minor matrilines and a single patriline [7, 16]. In addition to loss of genetic diversity, a severe imbalance in existing diversity was evident from PCoA and STR-associated DLA class I and II haplotype frequencies. PCoA plots confirmed that a large proportion of the population was more homogeneous with comparatively few genetic outliers. The relatedness of most Standard Poodles was also reflected by a lack of diversity in the STR-associated DLA class I and II regions. The two most common DLA class I haplotypes comprised 45 % of all recognized haplotypes and the four most common made up 90 %. A single DLA class II haplotype was present in a heterozygous or homozygous state in 83 % of the population, which was similar to our earlier study [7].

A conclusion of this study was that inbreeding resulting from artificial genetic bottlenecks such as the MCB resulted in the entrance of autoimmune diseases such as SA and AD into the breed. Genomic markers, when analyzed for % observed heterozygosity, showed that the subpopulation of dogs with SA were less heterozygous than either healthy control dogs or AD dogs. IR values confirmed that dogs with SA were indeed more inbred as a whole than healthy dogs or dogs with AD. However, there was no difference in the healthy and AD populations based on either % observed heterozygosity or IR values. PCoA based on genomic markers placed dogs with both SA and AD in close association with each other and with the main and most inbred population of Standard Poodles. These findings indicated that the genetic traits responsible for SA are not completely fixed in the 70 % of the poodle population that is at greatest risk, while the traits responsible for AD are probably fixed.

Pedigree analysis also linked both SA and AD with the MCB and a rapid rise in COIs starting in the 1960s and peaking in the 1980s. The COIs based on deep pedigrees decreased thereafter, possibly because more and more breeders acted on Armstrong’s admonition to choose mates more carefully to lower COIs [17]. The relatively recent increase in % OEA over the last two decades may reflect attempts to lower the % MCB and % Wycliffe. Although increasing COI was associated with an increased incidence of SA and AD in the population, the effect of recent decreases in COI on these diseases is unknown, as no accurate incidence figures have been compiled. Anecdotally, Standard Poodle breeders believe that the incidence of SA is decreasing in the 2010s, although the situation for AD is more uncertain.

Although pedigree studies indicate that both SA and AD are associated with the MCB, they do not answer the question as to whether the causative traits were ancestral or new. The fact that SA and AD occur so commonly in a number of seemingly unrelated breeds strongly favors an ancestral origin for both disorders [13, 14, 33]. Pedigrees coupled with disease histories also suggest that the genetic traits responsible for SA and AD entered the MCB through different lineages and at different times and that the incidence increased rapidly when the genetic contributions of those ancestors reached a critical threshold or tipping point. In this scenario, the MCB was the force that both disseminated and concentrated the genetic polymorphisms responsible for increased SA and AD susceptibility.

This study also attempted to identify a significant association between specific STR-associated DLA class I or II haplotypes and either SA or AD. Given the number of dogs to be tested, a decision was made to use STRs to determine DLA class I and II haplotypes rather than sequencing. Although, DLA class II haplotypes are easily defined by sequencing polymorphic regions in exon 2 of DQB1, DQA1 and DRB1 genes [34], it does require time and expense, particularly when done on many hundreds of dogs. Identifying DLA class I haplotypes is much more difficult, time consuming and expensive, even with more improved techniques [35]. The GC richness in the DLA88 gene affects the efficiency of priming and amplification and there is a need for extensive cloning. Therefore, there was a distinct advantage in using linked STR markers for both DLA regions. DLA-linked STRs reflect the genetic makeup within its position on the genome, regardless of structural factors that limit PCR amplification and sequencing. As expected, there was strong linkage between specific STR alleles and DLA class I and II haplotypes. Given the strong linkage disequilibrium inherent to the DLA region [6, 7, 16], the occurrence of extended haplotypes between STR-associated DLA class I and II haplotypes was expected. The relatively high incidence of recombination between the DLA class I and II regions, which might not be expected, can be explained by recombination hotspots that exist in the major histocompatibility complex [36].

Several moderate protective and disease susceptibility associations were found between specific DLA Class I haplotypes and SA or AD. Only the STR-associated DLA class I haplotype 1003 was significantly associated with SA and AD at a low to moderate RR of 1.63 and 1.43, respectively. There was also a moderate protective association with having any of the minor class I haplotypes 1008–1057 versus the major haplotypes 1001–1007. At least two DLA class II haplotypes 2004 and 2006, conferred 2.4–2.8 times greater risk for develop AD (*p* = 0.01) or SA (*p* = 0.009). Homozygosity among these DLA haplotypes did not further enhance risk, which was also observed in an earlier study of SA in Standard Poodles that interrogated the entire DLA with SNPs [7]. The fact that SA and AD were associated with different DLA class I and II haplotypes supports what is observed in humans, i.e., that predisposition for autoimmune disease may involve many regions of the genome, but the clinical form that it takes is often determined by the HLA [20].

Although certain DLA class I and II haplotypes conferred an increased risk for AD and SA, or were protective, they only occurred in 3–9 % of the population. There is some question, therefore, as to the actual nature of DLA class II haplotype associations with various autoimmune diseases in dogs [37]. The entire DLA region is under strong linkage disequilibrium [6, 7, 16] and inbreeding can result in disproportionately fewer and fewer DLA haplotypes as more and more diversity is lost. If, as the present study demonstrates, autoimmune disease becomes more prevalent in the most inbred portions of the population, it follows that certain DLA class I and II haplotypes could undergo either real or inadvertent positive selection and become either a cause or marker of autoimmunity. If real, associations based solely on relative haplotype frequencies in healthy and diseased dogs should be confirmable by genome wide association studies (GWAS). No significant association with SA was found in the DLA region of Standard Poodles using single nucleotide polymorphisms (SNPs) from GWAS [7]. In contrast, the strong DLA class II association observed in Pug dogs with NME was confirmed by a significant hits in the DLA class II region by genome wide association studies (GWAS) using both STRs [38] and SNPs [39]. GWAS also implicated the DLA in pancreatic acinar atrophy in German Shepherd dogs [13] and later confirmed by sequencing [40]. In contrast, a DLA class II haplotype association in anal furunculosis in this same breed could not be confirmed by GWAS [40]. Genome wide association studies have also failed to confirm DLA association in lupus-like disease in Nova Scotia Duck Tolling Retrievers [8].

Controversy has existed from the onset of closed registries over the effects of inbreeding in dogs, as it has been with humans, other animals and even plants. However, inbreeding has occurred to some degree in dogs since a species of small wolves attached their evolutionary fate to humans as long as 40,000 years ago [41]. This initial attachment undoubtedly involved genetic adaptation of a Darwinian nature, and even though environmental pressures led to non-random selection to increase fitness for a new life-style, it was natural and not man-made. Positive selection undoubtedly accelerated as dogs crossed the threshold from camp followers to camp participants and was more likely to involve human rather than natural selection [16]. Further non-random and human driven selection can be attributed to the Neolithic period when people settled into an agricultural and pastoral lifestyle and dogs integrated with village life [42]. Dogs from this Neolithic expansion became the village dogs and landraces of the world, and individuals from these populations were selected for their abilities to perform specific tasks, including hunting in various forms, guardian of home or flock, and simple companionship. The effect of this gradual inbreeding over thousands of years on the health of *Canis familiaris* has gone largely unappreciated. However, it is evidenced by the wide range of heritable disorders that plague modern dogs, regardless of whether they are random-, mixed-, or pure-bred [43]. Although these ancient heritable diseases are troublesome, they have become more or less accepted. An even more profound effect of inbreeding has occurred since the Victorian era and the creation of registries that codified specific breeds and closed populations from outside genetic introgressions. This subsequent period of intense inbreeding has not only increased the incidence of ancient diseases, but it has also led to a great increase in simple Mendelian diseases. These simple, and almost exclusively recessive, deleterious traits are much more likely to be seen in purebred dogs, and many are breed specific. Some of these recessive mutations are also ancient, but never of consequence given their previously low frequency in the population. Many of these deleterious recessive traits are new. In either case, inadvertent positive selection has brought them to the forefront. They often occur in complex genetic pathways affecting organs such as eyes, heart/blood, muscle, or nervous system with some regularity [44]. They have been documented in 500 known dog breeds and new ones are published and catalogued monthly [45]. This large array of continually evolving genetic diseases has even promoted the use of purebred dogs for studies of corresponding diseases in humans [46].

One of the objectives of this study was to provide Standard Poodle breeders with the genetic information required to manage diversity within the breed and improve its health. This study implicates past and present inbreeding as a major risk factor for diseases such as SA and AD. Therefore, increasing genetic diversity across the breed should decrease the incidence of these diseases, as well as other heritable disorders. Given the long history of transoceanic interbreeding of Standard Poodles, geographic separation is not a reliable predictor or source of genetic diversity. Genetic diversity can be measured by both pedigrees and genetic tests based on DNA. Pedigrees are valuable in calculating indices such as % MCB, tracing ancestors that may have been sources of heritable diseases, or calculating COIs. However, they must be deep enough to span important genetic bottlenecks or founder effects. Moreover, pedigrees measure theoretical genetic contributions of ancestors, while tests for genetic markers measure the actual contributions. Therefore, tests based on genetic markers should be used to complement and enhance pedigree data. The primary goal of genetic testing should be to identify genome-wide diversity and to use this information to increase, rebalance or introduce new diversity. Maintaining genetic diversity in the DLA region is also important for balancing self and non-self antigen recognition, although assessing and regulating genome-wide diversity purely through DLA markers is genetically flawed. Standard Poodle breeders may have several strategies to confront their genetic diversity problems [47]. The simplest strategy may be to rebalance the genetic diversity that still exists across the breed. This can be done by increasing the contribution of genetic outliers, which are a minority of the population but contain a majority of the genetic diversity. Although this study tried to identify as much existing diversity among Standard Poodles as possible, more genetic diversity may still exist and should be sought out. Diversity could also be increased by bringing in entirely new blood, such as the outcrossing of Standard Poodles with Miniature Poodles. Although Miniature Poodles are genetically distinct from Standard Poodles [54], poodles are registered by size and such crosses are allowed by the AKC and some other registries but are not widely accepted. Although Miniature Poodles can add a significant additional pool of genetic diversity to Standard Poodles, there are also similar breeds that could be used for outcrossing. However, this must be done with great care to preserve and distribute new diversity and to prevent the introduction of new heritable disease traits. Finally, research must continue to identify specific causes and genetic markers for heritable diseases that currently affect the breed’s health, such as SA and AD.

## Methods

### Sample collection

The 761 dogs in this study included Standard Poodles from Europe (*n* = 57), USA (*n* = 478) and Canada (*n* = 138), as well as Miniature Poodles (*n* = 16) and Standard Poodle × Miniature Poodles (*n* = 72). Most of the dogs were from common conformation lines, while others were from obscure bloodlines that had been identified. Certain Miniature Poodles are being used for outcrossing to Standard Poodles and some of these dogs along with Standard Poodle × Miniature Poodle offspring and their backcrosses were included. Sixty one Standard Poodles in the study, all from the USA, suffered from SA and 74 from AD. Samples were submitted with a form listing details of the dog’s pedigree and health. Samples were collected under UC Davis IACUC protocol #16643.

### DNA extraction

DNA was extracted from both buccal swabs and EDTA treated whole blood. DNA was extracted from a single cytology brush by heating at 95 °C in 400 μl 50 mM NaOH for 10 min and the pH neutralized with 140 μl 1 M Tris–HCl, pH 8.0 [24]. Blood samples (200 μl) were extracted using QIAGEN QIAamp®DNA blood mini and midi kits (QIAGEN Inc., Valencia CA, USA).

### Genetic diversity testing

Thirty three STR loci from across the canine genome were multiplexed into two panels (Table 1). One panel included 20 of 21 di-STRs recommended for canine parentage verification by the International Society of Animal Genetics (ISAG) [48]. Amelogenin gene primers for gender determination were also included [49]. A second panel consisted of two additional di-STRs, FH2001 and LEI004 and 10 of 15 tetra-STRs validated for forensic testing [50]. Primers, dye labels, repeat motif, allele size range and known alleles for this set of markers can be found in the preceding references and in Pedersen et al. [6]. Genotyping was conducted by the Veterinary Genetics Laboratory, UC Davis, and data were analyzed using STRand software [51].

### Determination of DLA class I and II haplotypes

Four dinucleotide STRs from regions flanking the DLA class I (*DLA88*) and three STRs associated with DLA class II (DLA-DRB1, −DQA1, −DQB1) were identified on Dogset [52]. Locus designations, primer sequences, number of alleles and allele size ranges are listed on Table 9. Specific alleles at each of DLA class I and II STR loci were found to be strongly linked, forming distinct haplotypes as determined by analysis with Phase [53]. Further Phase analysis identified strong linkages between DLA class I-II haplotypes that proved helpful in correcting errors made by independent Phase analyses of each region. Specific STR-associated DLA class II haplotypes corresponded to officially designated sequence based haplotypes, as determined by doing STR based testing of DNA on several breeds of dogs [6, 16] in addition to Standard Poodles [7] and Miniature Poodles [54] that had been previously tested for exon-2 based DLA class II haplotypes. The association between STR and exon-2 sequence based DLA class II haplotypes has been also validated in Italian Greyhound [55].

**Table 9:** Primer sequence and allele information for STRs in the DLA region of Poodles

	ID	Forward primer	Reverse primer	# Allele	Size range
DLA-I	3CCA	TGTACTTGAACACTCCCTGCAC	CCTCACATAGCCCTTCTCAGC	14	375–393
	4ACA	CAAAGGAAAGCCTTTGAGTTCG	GCTGGGCCCATACCTCATCT	12	364–381
	4BCT	ATGCCAGAGGTTGGAGCATT	GGAGCAGAGAAAACAAGCGAAT	6	277–293
	1131	TTGTGCCCTCAGGAAAAACC	GGGGCATTGTGGAGTAGAGC	9	178–188
DLA-II	5ACA^a^	TATTGCACCCTGGTGTCTGC	GTTTCTT TTGCCCTGGGTGGTAAAATC	6	339–351
	5ACT^a^	GGAACCCCCTGTAAAATTTCTT	GTTTCTT CAGCCAAGACCTTAGGAGCAA	7	321–329
	5BCA^a^	GTTTCTT CCCTGGATATGTGGCAGTCA	TGCCCTCTTCCACTTCACCT	6	268–288

^a^These primers have 7 bp pigtails

### Statistical analyses

Genetic diversity estimates were calculated from allele and allele frequency data from 33 genomic STR loci using GenAIEX 6.5 [56]. Principal coordinate analysis was also done with GenAIEX 6.5. Relative risk (RR) was determined by the MedCalc calculator [57]. All possible pair-wise comparison was performed by TukeyHSD with 95 % confidence interval in R.

Internal relatedness (IR) reflects the relationship of an individual’s parents as described by Amos et al. [27] and based on an earlier calculation by Queller and Goodnight [58]. IR is a measure of heterozygosity that weights allele sharing by allele frequency and is highly correlated with standardized heterozygosity and with heterozygosity weighed by locus [32]. IR values were calculated for accidental full-sibling breeding of Standard Poodles and the average IR for the offspring was 0.25, rather than the anticipated value of 0.5 predicted by the published equation [27]. Therefore, an IR value of ≥0.25 was used to measure relatedness equivalent to or greater than what would be expected among offspring of full-sibling pairs.

IR values were graphed in two manners: 1) comparing individual Standard Poodles with other poodles in the population, and 2) comparing the frequencies of STR alleles in individual Standard Poodles with the frequency of the same alleles in village (indigenous) dogs. The second comparison accounted for potential loss of diversity that occurred as a result of breed development since the registry was established in 1874 in England. Village dogs from the Middle East, SE Asia and Pacific region are the most outbred population of dogs studied and have been used as a genetic gold-standard for modern breeds [16].

### Pedigree analyses

The Standard Poodle Database [59] currently contains 235,351 pedigrees that go back to the establishment of registries. This database is widely used to COIs and relationship as originally described by Wright [60]. Coefficients of inbreeding were calculated using PedScope (Tenset Technologies LTD, Cambridge, UK). The % MCB, Wycliffe, and OEA were calculated using the Standard Poodle Database and a program developed by one of the authors (LB) and run under Paradox [18]. Pedigrees are accessed for each dog in a specific database and analyzed starting with the founders and working forward to the present. Relationship coefficients were then computed for each dog. The % Wycliffe is based on ancestry to five dogs, Sedberght Mitzi, Annsown Gay Knight, Petticote Domino, Carrillon Michelle, and Carillon Dilemma. The % MCB is calculated from ten dogs - Annsown Sir Gay, Beltore Bright Star, Lowmont Lady Cadette, Canorwoll of Thee I Sing, Carillon Michelle, Robin Hill of Carillon, Petitcote Domino, Petitcote Bubbling Over, Clairedge Cinderella, and Bel Tor Hosanna. The seven dogs used to calculate % OEA included Vulcan Golden Light, Alpenden Owstonferry Golden Orial, Pinetum Shantung Tatters, Frenches the Golden Horn, Tangerine of Whittens, Vulcan Merry Sonatina, and Vulcan Champagne Tansy.

The ranking of individual dogs on an influential ancestor list was generated using PedScope (Tenset Technologies, Ltd., Cambridge, UK) based on the methodology of Boichard et al. [61] and Lacy [62]. Coefficients of inbreeding for 10 and 15 generations were also calculated using PedScope.

### Data on SA and AD incidence

Based on pedigree and other records of contemporary dogs, many of which were included in this study, 1643 dogs were found to have no close relatives with AD or SA for three generations and were used as a healthy control group. Five hundred twelve dogs with deep pedigrees, including those in this study, were identified with AD and 465 with SA.

## Conclusions

We demonstrated that the incidence of both Addison’s disease and sebaceous adenitis in Standard Poodles increased as a result of an artificial genetic bottleneck involving a small group of show-winning founders from the mid-twentieth century. This bottleneck led to inbreeding over two decades with the results that 50–60 % of an average Standard Poodles ancestry can be traced to a few lines. We conclude that a number of ancestral traits associated with autoimmunity, some common to both SA and AD and some unique to each, were concentrated by inadvertent positive selection during close linebreeding for show winning form.
